# Template-Based Step Detection with Inertial Measurement Units

**DOI:** 10.3390/s18114033

**Published:** 2018-11-19

**Authors:** Laurent Oudre, Rémi Barrois-Müller, Thomas Moreau, Charles Truong, Aliénor Vienne-Jumeau, Damien Ricard, Nicolas Vayatis, Pierre-Paul Vidal

**Affiliations:** 1 L2TI, University Paris 13, 93430 Villetaneuse, France; 2 COGNAC-G (UMR 8257), CNRS Service de Santé des Armées University Paris Descartes, 75006 Paris, France; 3 CMLA (UMR 8536), CNRS ENS Paris-Saclay, 94235 Cachan, France; 4 Service de neurologie, Hôpital d’Instruction des Armées Percy, Service de Santé des Armées, 92190 Clamart, France; 5 Hangzhou Dianzi University, Hangzhou 310005, Zhejiang, China

**Keywords:** inertial measurement units, gait analysis, biomedical signal processing, pattern recognition, step detection, physiological signals

## Abstract

This article presents a method for step detection from accelerometer and gyrometer signals recorded with Inertial Measurement Units (IMUs). The principle of our step detection algorithm is to recognize the start and end times of the steps in the signal thanks to a predefined library of templates. The algorithm is tested on a database of 1020 recordings, composed of healthy subjects and patients with various neurological or orthopedic troubles. Simulations on more than 40,000 steps show that the template-based method achieves remarkable results with a 98% recall and a 98% precision. The method adapts well to pathological subjects and can be used in a medical context for robust step estimation and gait characterization.

## 1. Introduction

Human locomotion is a complex mechanism composed of a succession of strides, steps, and phases [1,2]. Some pathologies (such as Parkinson’s disease, arthritis, stroke, obesity, diabetes, etc.) may alter the locomotion, threatening the autonomy of patients and increasing the risk of fall. The objective quantification and assessment of locomotion is therefore a crucial problem, that has been addressed in the literature by measuring the movement with several types of sensors such as inertial sensors, instrumented mat, force platforms, camera-optical tracking system or force-sensitive resistors insoles. The signals obtained from these sensors are processed (automatically or manually) so as to extract some features that characterize the locomotion (speed, variability, smoothness, etc.). Computing these features can help to compare subjects or to provide a follow-up on a particular subject [3,4,5,6,7,8,9,10].

Since the breakthrough of low-cost sensors, numerous works have focused on the quantification and characterization of locomotion with Inertial Measurement Units (IMU) composed of accelerometers, gyroscopes and magnetometers. The main advantages of these sensors is that they are relatively low-cost, they do not require a dedicated room for the experiments, and their small size makes them easy to handle in day-to-day clinical situations. They have proven to give interesting results in several clinical studies in rehabilitation [2,11,12], neurology [13,14,15], orthopedics, physical therapy, functional electrical stimulation (FES) [16,17] etc.

Among the gait features that have proved their relevance in a medical context, several are linked to the notion of step (step duration, variation in step length, etc.), which can be seen as the core atom of the locomotion process [3,4,5,6,7,8,9]. Many algorithms have therefore been developed to automatically (or semi-automatically) detect the gait events (such as heel-strikes, heel-off, etc.) from accelerometer/gyrometer signals [4,6,7,11,12,13,14,15,18,19,20,21,22,23,24,25,26,27,28,29,30,31,32]. Most of the time, the algorithms used for step detection are dedicated to a specific population (healthy subjects [6,22,24,25,26,27,28], elderly subjects [3,19,20], Parkinson patients [33], etc.) and only a few publications deal with heterogeneous populations composed of several types of subjects [4,5,7,11,13,15,18,23]. Another limit to existing algorithms is that they often focus on locomotion in established regime (once the subject has initiated its gait) and do not deal with steps during U-turn, gait initiation or gait termination. Yet, initiation and termination steps are particularly sensitive to pathological states. For example, the first step of Parkinsonian patients has been described as slower and smaller that the first step of age-matched subjects [34]. U-turn steps are also interesting since 45% of daily living walking is made up of turning steps, and when compared to straight-line walking, turning has been emphasized as a high-risk fall situation [35]. This argues for reliable algorithms that could detect initiation, termination and turning steps in both healthy and pathological subjects.

The common approach for step detection is based on the use of filtering/thresholding/peak detection techniques [6,7,11,12,13,14,15,21,22,23,24,25,26,27,28,29,30,31,32] that are applied on the accelerometer/gyrometer signals. The aim is to recognize one specific event, supposedly characteristic of the step (such as a local maximum or the time when the signal exceeds a threshold). First, a preprocessing step is performed where the signal is filtered so as to emphasize the event that they seek to detect or to remove other events. The most well-known preprocessing stage was introduced by Pan-Tompkins [36] and is composed of several signal processing blocks (bandpass filtering, derivation, squaring, etc.). Designed at first for ECG signals, this preprocessing has been used in various step detection methods [11,21,29,30]. Wavelets transforms have also proven to give good preprocessing results [25]. After this possible preprocessing stage, the steps are detected with empirical or dynamic thresholds, peak detection methods, of a combination of both. Other methods seek to detect each phase of the walking process by using dedicated signal processing techniques (such as peak detection, zero-crossing, etc.). Unfortunately, all these methods heavily rely on the calibration of several parameters (width of the bandpass filter, window length, thresholds, etc.) which are difficult to estimate and thus often set according to empirical experience. This calibration might be tricky, especially if the algorithm is to be used on subjects with different pathologies or different degrees of severity [7]. In addition, most of the algorithms designed for a particular type of subject may suffer from degraded performance in other cohorts [11].

For these reasons, some articles have mentioned the use of templates for step detection [18,19,20,21,37]. In this context, a template can be seen as a typical step, whose characteristics (amplitude, shape, duration) are typical of a type of step. Existing step detection methods using templates have only investigated the possibility of using one [19,20,21] or two [18] templates. These templates can either be manually defined [20] or extracted from the data for one subject [19,21] or from a training database [18]. We propose to extend this idea by constructing and using a library of several templates, composed of a large set of typical steps that may be found in several pathologies or experimental conditions. The main intuition behind this is that it is a known medical fact that there exist several types of steps (according to interpersonal variability, age, speed and pathology). The existence of different types of steps is in fact a taxonomy based on the clinical examination of the patients as it developed throughout the past century in hospitals. In that sense, the use of templates can be seen as an attempt to emulate clinics. Therefore, it might be complicated to try to detect steps with one specific model (which is implicitly what is done when using one template or when using filtering/thresholding/peak detection methods that depend on one set of parameters, thresholds, detection criteria, etc.) In order to overcome this issue, it can be useful to use a library of models (in our case, a library of templates) which represent typical step cycles from healthy, pathological, young, elderly, etc. subjects. Hopefully, the use of this library can improve the robustness of the detection and paradoxically, prevent the overfitting induced by the choice of many parameters. This approach can also enable one to deal with complicated cases such as pathological steps, or steps that occur during initiation, U-turn or termination.

This article is organized as follows: Section 2 describes the data and the protocol, as well as the data annotation process. Section 3 describes the step detection method based on template matching. Section 4 presents the results of our method and a comparison to state-of-the art methods. Section 5 provides a discussion on the robustness of the method and several insights for the possible use of this algorithm in a clinical context.

## 2. Data and Experiments

### 2.1. Data Acquisition and Protocol

The data used for the conception and testing of the method presented in the article has been provided by the following medical departments: Service de chirurgie orthopédique et de traumatologie de l’Hôpital Européen Georges Pompidou, Assistance Publique des Hôpitaux de Paris, Service de médecine physique et de réadaptation de l’Hôpital Fernand Widal, Assistance Publique des Hôpitaux de Paris, Service de neurologie de l’Hôpital d’Instruction des Armées du Val de Grâce, Service de Santé des Armées. The study was validated by a local ethic committee (Comité de Protection des Personnes Ile de France II, CPP 2014-10-04 RNI) and both patients and control subjects gave their written consent to participate. All signals have been acquired at 100 Hz with wireless XSens MTw^TM^ sensors (autonomy 6 h, device dimension 47 × 30 × 13 mm, weigth 16 g, acceleration range ±160 m/s^2^, angular velocity range ±2000 deg/s, dynamic accuracy roll/pitch 0.75 deg RMS, dynamic accuracy heading 1.5 deg RMS) located at the right and left foot and fixed using a Velcro band designed by XSens^TM^. XSens sensors were chosen based on their relatively low cost, the availability of raw data as well as previous analysis that enlightened their widespread use in clinical settings [38]. As for the location of the sensors, we used data from a sensor placed on the dorsal part of one foot to detect steps based on previous work which proved high reliability and precise phase detection using such an apparatus [2]. One noticeable advantage of such a position is the possibility to use a second symmetrical sensor for direct back-up without changing the algorithm. Besides, as opposed to detection algorithm from lower back sensors, direct data are used (one step being defined as the foot—and not the trunk—displacement). For the routine clinical examination of patients, two other sensors were also placed in the back at L5 level and on the front of the head. These data, although of use for the description of the clinical syndromes we quantified, will not be considered further as this manuscript focuses on step detection.

The signals obtained with both sensors were automatically synchronized by the acquisition software. All subjects were asked to:

- stand quiet for 6 s
- walk 10 m at preferred walking speed on a level surface
- make a U turn
- walk back
- stand quiet 2 s

For practical reasons, patients kept their own shoes. Average speeds ranged from 1 m·s^−1^ to 1.8 m·s^−1^. The database is composed of 230 subjects who performed the protocol between 1 and 10 times, which totals 1020 recordings. The subjects’ characteristics are presented in Table 1. Healthy subjects had no known medical impairment. The orthopedic group is composed of 2 cohorts of distinct pathologies: lower limb osteoarthrosis and cruciate ligament injury. The neurological group is composed of 4 cohorts: hemispheric stroke, Parkinson’s disease, toxic peripheral neuropathy and radiation induced leukoencephalopathy.

**Figure 1:**
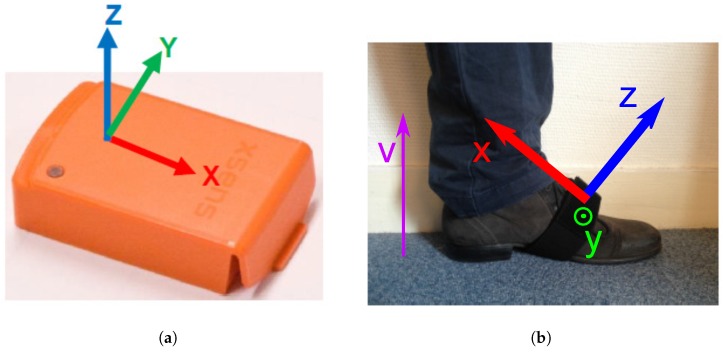
Presentation of the XSens^TM^ sensor. (**a**) XSens^TM^ sensor; (**b**) Definition of the axis for the XSens^TM^ sensor located at the left foot.

The protocol includes 2 sensors (left and right foot), and each of them records a 9-dimensional signal (3D accelerations, 3D angular velocities, 3D magnetic fields), possibly with some re-calibrated data provided by the XSens^TM^ software (such as the vertical acceleration in the direction of the gravity). Instead of considering all these dimensions, we decided to only use a subset of them, and select the most relevant in the context of step detection. This decision has been made based on observations of real data and physiological reasons provided by medical doctors (see Acknowledgments and [2,39,40]). We decided to only select the components that were most reflective of the locomotion process (see Figure 1 for the definition of the axis): the Z-axis acceleration, the recalibrated vertical acceleration (vertical movements of the foot) and the Y-axis angular velocity (swing in the direction of the walk).

### 2.2. Data Annotation

Several publications related to automatic step detection have provided a comparison of their results with existing systems such as 3D motion tracking systems [15,19,22,27], video camera [19,26], force platforms [26,27] or instrumented mat [5,7,20,24,25]. However, this comparison, while allowing an objective evaluation of the algorithm, is not easily feasible on a large scale due to practical reasons. Indeed, a strict comparison of our results with other types of step detection would require to test the six different cohorts of patients we have presently tested with IMUs with a 3D tracking system, which is not easily transported in various hospitals and impossible to use in the context of a daily clinical routine. Since the aim of this article is to provide a method that adapts well to different contexts and types of subjects and pathologies, we chose to use a light protocol that was compatible with clinical constraints, so as to maximize the size of the cohorts.

In that context, the evaluation of the method and the determination of the library of templates was therefore performed thanks to manual annotations provided by experts that were trained to deal with physiological signals. These experts have been trained on two gait validation tools (instrumented mat: GAITRite from CIR Systems and motion capture: CodaMotion) in a preliminary study, so as to learn how to annotate the signals. This learning phase was performed on five healthy subjects in laboratory conditions and the manual annotation error was calculated as 2 samples (std : 1.0) with CodaMotion and 1.7 samples (std : 1.2) with the GAITRite.

All steps were manually annotated by specialists using a software allowing to point with the mouse the starts (foot-flat) and the ends (heel-off) of the foot flat periods during which the sensor is not moving. The annotations were performed thanks to the Z-axis acceleration (normal to the upper foot surface) which is the most sensitive direction to detect the movements of the foot with respect to the floor. For the tricky cases of pathological gaits, a first gross annotation was made and then refined by zooming in on each step. The uncertainty of this annotation is evaluated to less than 0.2 s (20 samples) for each mouse click. In total, the database is composed of 40,465 steps (20,240 extracted on the right foot and 20,225 on the left foot). Even though they had a distinct shape, the U-turn steps were also taken into account. In the database, 2671 steps are considered as belonging to the U-turn.

**Figure 2:**
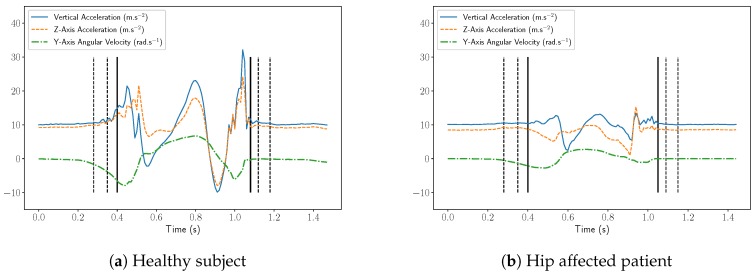
Vertical acceleration, Z-axis acceleration and the Y-axis angular velocity recorded from the right foot. The vertical dot lines display the different possibilities for start/end times and the plain lines display the choice made by the experts.

Examples of these 3 components (Z-axis acceleration, vertical acceleration and Y-axis angular velocity) recorded at the right foot are presented on Figure 2a,b for respectively an healthy and hip-injured patient. It appears in these figures that the amplitudes of the signals are clearly different and it is likely that classical threshold-based methods would hardly perform well on both subjects. However, the structure and shape of the step is roughly the same for both subjects so it might be relevant to use a template-base method. Nevertheless, these examples also display the main difficulties in conceiving an automatic algorithm for step detection:

- The uncertainties in the definition of the starts and ends of the steps. Indeed, we can see in Figure 2a, that many choices would be acceptable: depending on the considered definition, the results may be different.
- The variability of the step patterns according to the pathology, the age, the weight, etc. For example, on Figure 2b, the subject is dragging his feet, causing an abrupt change in the step pattern (noisy part at the end of the step).

## 3. Method

### 3.1. Library of Templates

The library of templates was manually chosen by the experts that made the annotations in order to cover most of the cases that were seen in the data. In total, 55 templates have been chosen by the experts. Among these 55 templates, 14 belong to healthy subjects, 12 to orthopedic subjects and 29 to neurological subjects. One template corresponds to a U turn step, 10 templates to initiation steps and one template to a termination step. The step durations go from 65 samples to 96 samples with an average of 76.9 samples.

### 3.2. Step Detection

The principle of our step detection algorithm is to recognize the steps in the signals thanks to a predefined set of templates. More precisely, our method uses a set of templates P: these templates have been manually extracted from real accelerometer data and checked by medical doctors and specialists of locomotion. Each template p∈P is a matrix of size $3\times N_{p}$ and corresponds to one step. $N_{p}$ is the number of samples of the template, and 3 is the number of components (vertical acceleration, Z-axis acceleration and Y-axis angular velocity).

These templates are to be compared to the signal we want to study by calculating some correlation coefficients. As the sequences we want to detect are variable in duration as well as in amplitude, we want to use a measure of fit that is independent of the scale but is able to identify the correspondences in shape. In addition, we want the comparison to be independent of the orientation of the sensor, so any DC component should be removed. In this context, it seems natural to use the Pearson correlation coefficient, which satisfies all these conditions, and is defined for two one-dimensional vectors *y* and *z* of length *n* as

(1) $$\rho \left(y,z\right)=\frac{\mathrm{cov}(y,z)}{\sigma_{y}\sigma_{z}}=\frac{E[\left(y-\mu_{y}\right)\left(z-\mu_{z}\right)]}{\sigma_{y}\sigma_{z}}$$

where $\left(\mu_{y},\mu_{z}\right)$, $\left(\sigma_{y},\sigma_{z}\right)$ are respectively the mean and standard deviation of *y* and *z*.

Let *x* be a multivariate signal, represented by a matrix of size $3\times N_{x}$: we want to detect the steps by using the set of templates P. Let us introduce the following notations:

- $N_{P}$ is the number of three-dimensional templates
- $N_{x}$ (resp. $N_{p}$) is the number of samples of *x* (resp. *p*)
- $x^{\left(k\right)}$ (resp. $p^{\left(k\right)}$) is the *k*th component of *x* (resp. *p*). In our case we have $k\in \left\{1,2,3\right\}$
- $x^{\left(k\right)}\left[t_{1}:t_{2}\right]$ is the portion of $x^{\left(k\right)}$ between time samples $t_{1}$ and $t_{2}$ (we therefore have $x^{\left(k\right)}\left[1:N_{x}\right]=x^{\left(k\right)}$)

The first step of the algorithm consists in calculating the Pearson correlation coefficients between the templates and the signal, for all possible time positions and all three components:

(2) $$\begin{array}{c} \forall k\in \left\{1,2,3\right\},\;\forall p\in P,\;\forall t\in 〚1,N_{x}-N_{p}+1〛\;r\left(k,p,t\right)=\rho \left(p^{\left(k\right)},x^{\left(k\right)}\left[t:t+N_{p}-1\right]\right) \end{array}$$

r(k,p,t) is the correlation between the *k*th component of template *p* and the *k*th component of the signal at time sample *t*. More precisely, we first pick one template *p* and one its component (for instance the Z acceleration $p^{\left(1\right)}$). This template is then slid along the corresponding component of the signal *x* (i.e., the Z acceleration $x^{\left(1\right)}$) and the correlation coefficient is computed for all lag positions. This process is then reiterated with the second and the third component, and then for each template. The algorithm runs until all correlations have been computed (all templates p∈P, all components $\in \left\{1,2,3\right\}$ and all lag positions $t\in 〚1,N_{x}-N_{p}+1〛$).

The second step is a local maxima search among the r(k,p,t) coefficients in order to extract the possible steps. r(k,p,t) is selected as a local maximum if it is greater than its nearest temporal neighbors. We define the set L of possible steps as:

(3) $$\begin{array}{c} L=\left\{(k,p,t)s.t.r(k,p,t)>r(k,p,t-1)\;\mathrm{and}\;r(k,p,t)>r(k,p,t+1)\right\} \end{array}$$

The set L contains all acceptable positions for the steps, and the coefficients r(k,p,t) with (k,p,t)∈L can be interpreted as the likelihood of having a step similar to the pattern *p* starting at time sample *t*.

**Figure 3:**
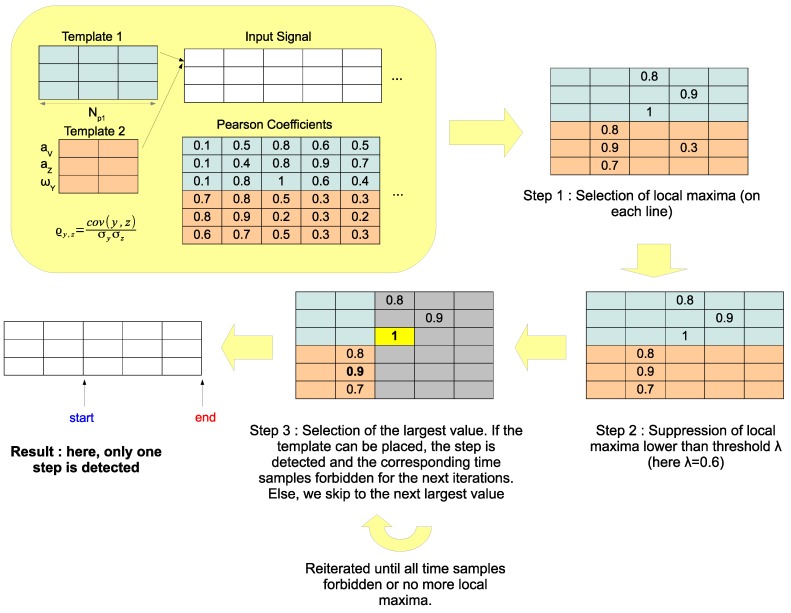
Chartflow of the step detection method.

Our step detection algorithm takes as input the set L and works as a greedy process. At each iteration, the largest value $r(k^{*},p^{*},t^{*})$ with $(k^{*},p^{*},t^{*})\in L$ is selected: if the step $p^{*}$ positioned at time sample $t^{*}$ overlaps with a previously detected step, it is discarded and we switch to the next largest value. Otherwise, if step $p^{*}$ can be positioned at time $t^{*}$, the step is detected and all time samples between $t^{*}$ and $t^{*}+N_{p^{*}}-1$ are forbidden for the next iterations. The process is stopped when all time samples are forbidden, when the set of possible steps L is empty, or when all values r(k,p,t) with (k,p,t)∈L are lower than a threshold λ. Note that in practice, the main purpose of threshold λ is to speed up the algorithm, as it reduces the size of set L. The algorithm is summarized in Algorithm 1 and illustrated in Figure 3.

A last post-processing step can be performed so as to discard the steps detected when the subject was actually not moving. These false detections occur when a fit is found with one template, even though the signal is almost equal to zero after DC component removal: this is in fact due to the invariance in scale provided by the Pearson correlation coefficients. A solution can be found by processing the final list of detected steps, and removing the steps whose standard deviation is way lower than the one of the template that was used for the detection. Formally, this step involves a threshold μ: given a detected step with start and end times $t_{start}$ and $t_{end}$, detected thanks to the pattern $p^{\left(k\right)}$, the step is to be discarded if

(4) $$\sigma_{x^{\left(k\right)}\left[t_{start}:t_{end}\right]}<\mu \;\sigma_{p^{\left(k\right)}}$$

where σ stands for the empirical standard deviation operator.

In the presented method, λ is set to 0.6 and μ to 0.1. The influence of these parameters will be described in the Discussion section.
**Algorithm 1:** Step detection algorithm. 
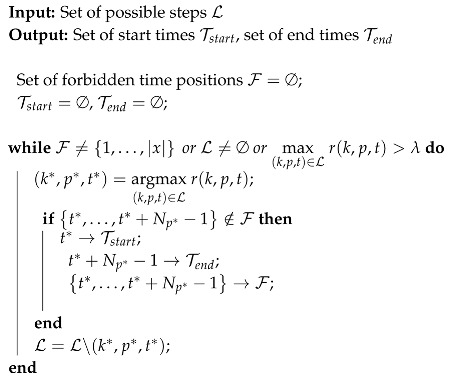



## 4. Results

This section presents the results of the template-based method and of a state-of-the-art method on the database composed of healthy, neurological and orthopedic subjects.

### 4.1. Evaluation Metrics

The following precision/recall metrics are used for the evaluation of our method based on the annotations provided by the specialists.

- **Precision (or positive predictive value).** A detected step is counted as correct if the mean of its start and end times lies inside an annotated step. An annotated step can only be detected one time. If several detected steps correspond to the same annotated step, all but one are considered as false. The precision is the number of correctly detected steps divided by the total number of detected steps.
- **Recall (or sensitivity).** An annotated step is counted as detected if the mean of its start and end times lies inside a detected step. A detected step can only be used to detect one annotated step. If several annotated steps are detected with the same detected step, all but one are considered undetected. The recall is the number of detected annotated steps divided by the total number of annotated steps.
- **ΔStart.** For a correctly detected step, it is the difference between the detected start time and the annotated start time.
- **ΔEnd.** For a correctly detected step, it is the difference between the detected end time and the annotated end time.
- **ΔDuration.** For a correctly detected step, it is the difference between the duration of the detected step and the duration of the annotated step.

### 4.2. State-of-the-Art

The reference procedure for step counting/detection is based on the Pan-Tompkins method [36]. First intended for ECGs, it was later adapted to detect steps in the vertical accelerometer signal [11,21,29,30]. It is composed of several successive signal processing steps, which are designed to emphasize the structure of the step, making it easier to detect. These steps can be summarized as:

- Bandpass filtering (between $f_{min}$ and $f_{max}$): removes the gravity component and the noise.
- Derivation: amplifies the slope changes in the filtered signal. Whenever the foot rises from the ground or the heel hits the ground, the acceleration slope changes significantly and it translates into a burst in the filtered signal.
- Squaring: makes all points positive and enhances the large values of the filtered signal.
- Integration: the signal is smoothed using a moving-window integrator of length $N_{inte}$.
- Peak search procedure: originally, Pan & Tompkins [36] used a threshold to find the phenomena they were looking for in the heart rate signal (every time the filtered signal was above the threshold, it was considered as detected). When they adapted the algorithm to the step detection problem, Ying et al. [21] relied on the fact that the filtered signal showed great regularity: a small peak was always followed by a bigger one (respectively matching the foot lift and the heel strike). The time span of the second peak was defined as the peak-searching interval on the real acceleration signal. The maximum on that interval was considered a step.

Note that this step detection procedure only allows to detect steps but not to precisely know the start and end times of the step. In addition, this method is not designed to perform properly during periods of no activity. We therefore added a post-processing step, which, once a step is detected, compares the standard deviation of a neighborhood around the detected peak to a noise level. The size of the neighborhood, as well as the noise level, are optimized by grid search so as to give the best performances.

In Ying et al. [21], the parameters used are $f_{min}$ = 0 Hz, $f_{max}$ = 20 Hz, $N_{inte}$ = 0.1 s. The peak search procedure is performed sequentially: they select one peak every other peak, starting with the second one. With these parameters, we obtain from our database a recall of 99.53% and a precision of 51.20%. In fact, the peak-search procedure is not adapted and tends to detect several peaks within a step except for one. This phenomenon has already been described by several authors [29,30].

In order to objectively compare our method to the Pan-Tompkins, we tested several values for $f_{min}$, $f_{max}$ and $N_{inte}$, as well as a more relevant peak-search procedure, which only selects the local maxima among the detected peaks, thus preventing multiple detections. In total, 5 parameters need to be optimized by grid search (filter bandpass × 2, integration window, neighborhood size and noise level). The results presented for this method are therefore optimized on the whole database so as to maximize the F-measure.

### 4.3. Results

The average recall for the template-based method is 98.34% and the average precision is 98.30%. For the Pan-Tompkins method, the average recall is 97.82% and the average precision is 95.72%. Detailed results by group of subjects are presented in Table 2. Differences between the two methods are significant (signed-rank Wilcoxon test, *p*-value < 0.01) for all groups and all metrics except for recall in the Healthy group (see Table 2). For the template-based method, the differences between the groups are all significant (Mann-Whitney rank test, *p*-value < 0.01) except for the precision between the Healthy and Orthopedic groups and the precision between the Orthopedic and Neurological groups. For the Pan-Tompkins method, the differences between the groups are all significant (Mann-Whitney rank test, *p*-value < 0.01) except for the recall between the Healthy and Orthopedic groups and for the precision between the Orthopedic and Neurological groups.

The performances of our method vary according to the type of steps (initiation/termination, U-turn or normal steps): detailed results by types of steps are presented in Table 3. The best results are obtained for normal steps (99.58% recall and 99.04% precision) and the worst obtained for U-turn steps (83.87% recall and 90.76% precision). The same situation is observed for the Pan-Tomkins method (95.59% recall and 98.86% precision for normal steps) with a large drop in the precision for U-turn steps (88.12% recall and 50.51% precision). Detailed results

**Figure 4:**
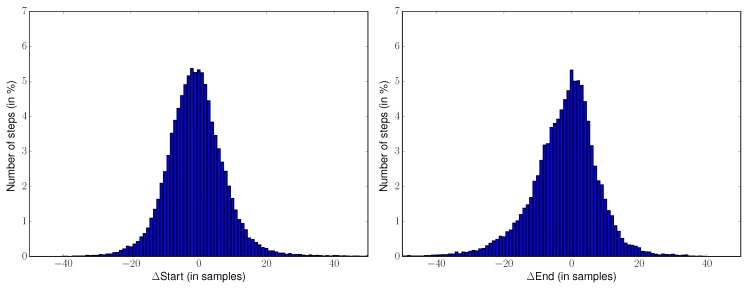

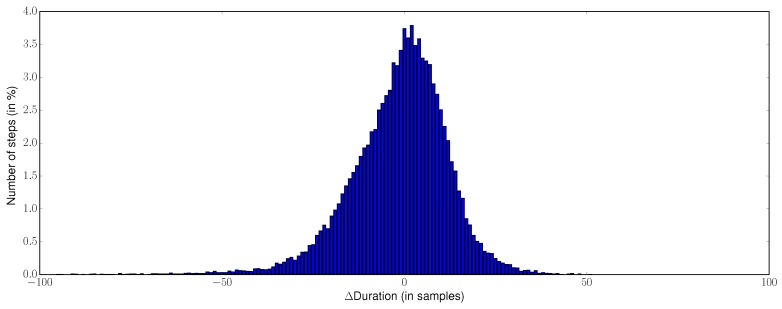
Differences between detected and annotated times ΔStart, ΔEnd, ΔDuration for the template-based method. The histograms are computed from the 39,706 steps that are correctly detected by the template-based method.

For all 39,706 steps that have been correctly detected by the template-based method, the average ΔStart is −0.7 samples (std : 9.4 samples), the average ΔEnd is −2.1 samples (std : 10.3 samples) and the average ΔDuration is −1.4 samples (std : 13.5 samples). Metrics are not available for the Pan-Tompkins method since it does not retrieve the start and end times of the steps. The histograms or the metrics ΔStart, ΔEnd, ΔDuration for all the correctly detected steps (39,706 here) for the template-based method are presented in Figure 4.

The median absolute errors for the start times, end times and durations are displayed in Table 4. For standard configurations (healthy subjects and normal steps), these median errors range from four samples (0.04 s) to seven samples (0.07 s). However, for complex cases such as U-turn steps or pathological subjects, the median error can increase up to 15 samples (0.15 s).

**Figure 5:**
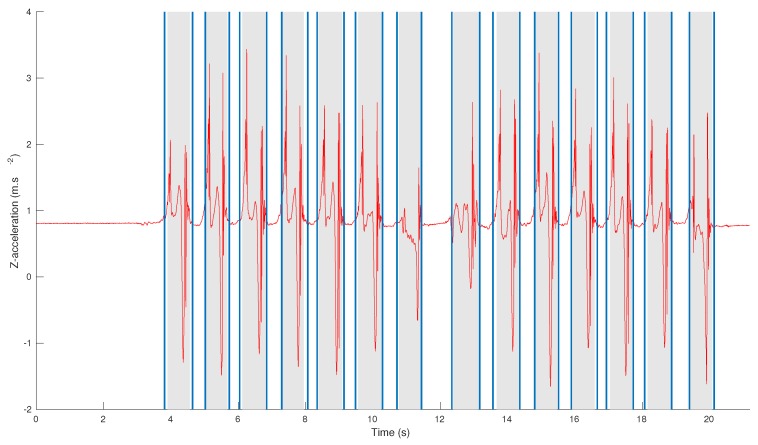

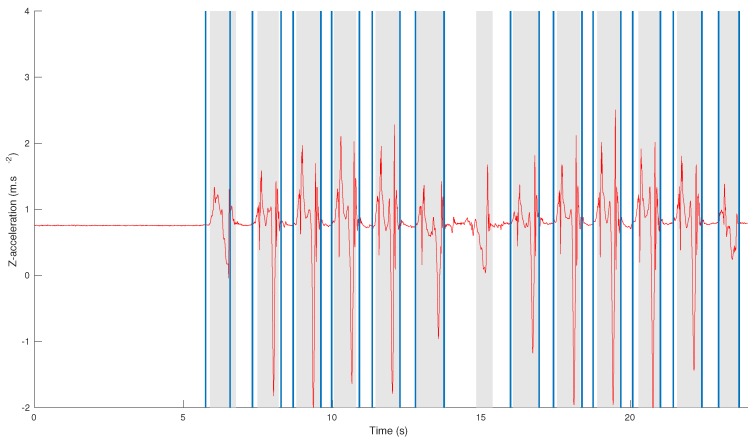
Comparison between step detection and annotations for a healthy subject (**top figure**) and a patient with osteoarthritis (**bottom figure**). The annotations are displayed as gray area and the step detection results as lines.

Examples of step detections obtained by our algorithm for a healthy subject and an osteoarthritis patient are displayed in Figure 5.

**Figure 6:**
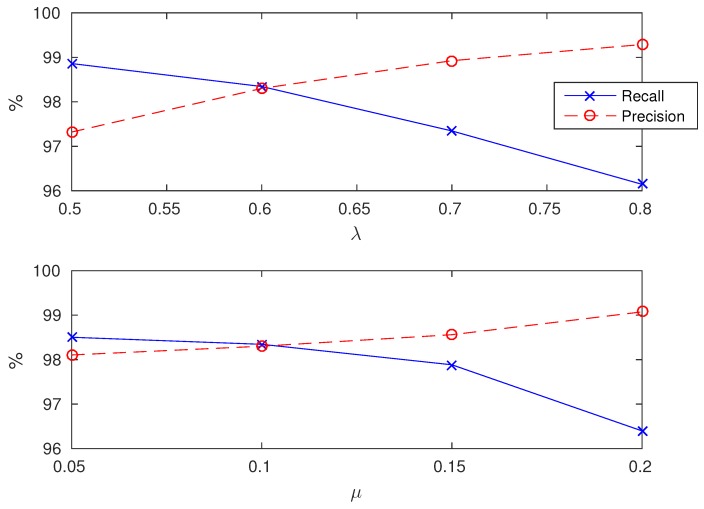
Influence of λ (**top figure**) and μ (**bottom figure**) on the precision and recall of the method. In the top figure, μ is set to its default value μ=0.1. In the bottom figure, λ is set to its default values λ=0.6

The algorithm is based on two parameters λ and μ that control the speed of the algorithm and the outlier rejection. The influence of these parameters on the results are displayed in Figure 6.

## 5. Discussion and Perspectives

The results presented in Table 2 show that the scores of the template-based method are consistent on all groups of subjects. The best performances are obtained for healthy subjects, but there is no large drop between the groups. This clearly shows that the method adapts well to different types of pathologies. One interesting observation is that the precision and recall of the method are well balanced, and the standard deviations of the scores are always lower than 4%. The results obtained with Pan-Tompkins are in the same order of magnitude but a different phenomenon seems to occur. While the scores are comparable with our method on healthy subjects, it is noticeable that the Pan-Tompkins method has difficulty dealing with neurological and orthopedics subjects. In particular, on these subjects, an over-detection occurs, thus decreasing the precision. One possible explanation is that signals associated to pathological subjects tend to have smaller amplitudes and to be noisier than those belonging to healthy subjects. Thus, if the parameters of the filtering are unadapted, the preprocessing tends to increase the level of noise and to create artifacts that are mis-detected as steps. This may be one limit of step detection methods based on signal processing: if the signals to be studied have different properties (noise, frequency content, amplitudes), it is tricky to find one unique processing adapted to all signals. This problem is overcome in template-based methods which inherently consider several models. The main idea behind the algorithm presented in this article is that there is not one typical step but rather several typical steps. This assumption is confirmed by the results obtained with state-of-the-art methods, which inherently define only one model and obtain degraded performances when confronted with pathological data.

The type of step (initiation/termination, U-turn, etc.) also impacts the detection results. As seen in Table 3, regular steps are more likely to be correctly detected. For both our method and the Pan-Tomkins approach, it appears that normal steps are easier to detect but their detection is also more robust than those of other steps (the standard deviations are at least three times smaller for normal steps). For initiation and termination steps, the performances do not drop but the results are more erratic than for normal steps. This suggests that in most cases, these steps are similar to normal steps, which allows to keep acceptable performances. However, for some patients with degraded gait, the initiation/termination steps strongly differ, and may not be detected, which causes the increase in the standard deviation. In addition, note that since the number of initiation/termination steps is small, the precision/recall is assessed on only 1 or 2 steps, so it is natural that the variations are large. The most challenging steps are the U-turn steps. For our method, the recall decreases because of the low amplitude of these steps, that are sometimes discarded in the last step of the algorithm because they are mistaken as outliers. For Pan Tomkins, an over detection appears during the U turn, due to the presence of several peaks in the signal that are mistaken as heel-strikes. Although both methods struggle with these steps, the Pan Tomkins strategy is here irrelevant, with a precision decreasing as low as 50%.

Interestingly, it appears from Table 2 and Table 3, that the differences between our method and the Pan Tomkins approach are more visible for complicated cases (pathological subjects and irregular steps). For these steps, standard signal processing procedures perform less accurately than for standard gait condition. In fact, it is difficult to tune the various parameters (filters, thresholds) so as to have them perform accurately in all situations. We believe this could also be the reason why the detection of these steps is less covered in the literature.

The repartition of the time estimation errors on all 39,694 correctly detected steps is presented in Figure 4. One interesting result is that our method does not introduce a bias: the average of the differences for all times (start, end, duration) is approximately equal to zero, and the distributions are approximately symmetric (skewness for ΔStart : 1.7, ΔEnd : −1.0, ΔDuration : −0.9). This tends to prove that the library is able to accurately detect the step boundaries and to adapt to various step durations. For 90% of the correctly detected steps, the errors for start and end times are lower than 0.15 s (in absolute value) and 0.21 s (in absolute value) for duration. These results are satisfactory when compared to the annotations uncertainties of experts and specialists, which are around 20 samples (0.2 s—see Section 2.2). Outliers are in fact due to two specificities of the database: tiny steps (under 50 samples) mainly located during U- turn (causing underestimation for start times and overestimation of end and duration times), and highly pathological steps for stroke subjects whose duration exceeds one second (causing upper outliers for start times and lower outliers on end and duration times). The method tested here is using templates of durations between 65 and 96 samples (see Section 3.1) and the detection is inevitably constrained by these step durations. While this phenomenon does not penalize the results on most steps, it is one limit of the algorithm. Should these outliers become more frequent, one possible solution is to increase the number of templates and to add typical steps corresponding to these outliers within the library.

The median absolute errors displayed in Table 4 confirm these assumptions. For standard conditions (healthy subjects and normal steps), the error is around four samples (0.04 s) for start/end times and seven samples (0.07 s) for durations. These values are coherent with state-of-the-art results that, depending on the publication, report gait events detection errors from 0.02 s to 0.06 s [11,19,24,25,41]. For normal steps and pathological subjects, the errors are still limited and compare with state-of-the-art which go from 0.06 s to 0.1 s [42,43]. For complex cases (especially U-turn steps), the error increases up to more than 10 samples (0.1 s) since the frontiers of the steps are more difficult to estimate and the amplitude of the steps is lower. As no algorithm in the literature currently deals with all these types of steps and such heterogeneous populations, we believe that the results on U-turn, although less precise than for normal steps, are still promising.

Figure 5 shows examples of step detection for a healthy and a pathological subject. For the healthy subject, the detection is very similar to the annotations with only a few samples of difference (for example on the first step). For the pathological subject who suffers from osteoarthritis, the shapes of the initiation and U-turn steps differ from the standard shape of the steps. For these reasons, the algorithm still detects the first step but is less precise for the start/end times. The U-turn step is not detected by the algorithm as its shape is unusual and its amplitude is too low. In fact, it is likely that the patient (who suffered from severe gait troubles and obesity), dragged his foot during the U-turn, and did not perform a proper step. In particular, his foot did not leave the ground, thus making it very difficult to detect this phenomenon with accelerometers. Thankfully these cases remained rare in the database, and U-turn steps still had an 83% average recall. Nevertheless, future works will investigate these situations by conceiving dedicated templates.

The algorithm uses two parameters λ and μ and one library of templates. Parameter λ is a threshold that allows to speed up the algorithm by discarding step matches that are unlikely to appear in the signal. Parameter μ is used in the postprocessing step in order to remove steps with low amplitude that may be due to noise and may cause false detection. The influence of both these parameters are displayed in Figure 6. It appears that these parameters mostly control the tradeoff between precision and recall. When λ is too large, the algorithm goes faster but discards true steps. When μ is too large, all low amplitude steps are discarded but true steps may be as well. However, all precision/recall values obtained with λ∈[0.5,0.8] and λ∈[0.05,0.15] are almost the same, which shows that a fine tuning is not necessary to make the algorithm work.

Intuitively, the composition of the library is a fundamental feature of the algorithm. The choice of the templates to be used is an interesting question that can be answered in many different ways. In a medical context, templates can, for example, be introduced according to the characteristics and pathologies of the subjects to be studied: a neurologist may benefit from a library of templates composed of a selection of different neurological pathologies. They can also be specified by experts such as biomechanists who can extract typical steps covering the whole range of types of locomotion (this is the approach we used in this article). Unsupervised machine learning techniques (such as dictionary learning) can also be used to automatically extract typical steps that are found on several exercises. It is also relevant to test semi-supervised techniques that could automatically choose the best library according to the input signal. All these leads are to be studied soon in collaboration with medical doctors and experts, and on more pathologies.

The aim of this study was to conceive an algorithm that only required data from small wireless sensors, and was able to perform robustly in the field, for various types of steps and heterogeneous populations. These constraints have led to the use of an adaptive methodology, that, instead of increasing the precision for standard conditions, sought to handle types of steps and cases that have rarely been addressed in the literature. The algorithm is usable in real clinical conditions and can provide results in all situations (degraded gait, U-turn, etc.). The method can also be used in offline software solutions, thanks to the low computational cost of the algorithm. Indeed, the processing of the whole database (more than 8.5 h of signals) takes less than 30 s.

## 6. Conclusions

We described in this article a template-based method for step detection. This method, based on a greedy algorithm and a library of annotated step templates, achieves good and robust performances even with a small number of templates. When used on a large database composed of healthy and pathological subjects walking at different speeds, the method obtains a 98% recall and 98% precision. Moreover, the algorithm allows one to detect the start and end times of each step with an acceptable precision, even on pathological subjects.

Thanks to its robustness and low computational cost, this method could be extended to process signals acquired in free-living conditions. Indeed, the actual protocol is composed of a no activity period and a U-turn, and there are no obstacles for testing the algorithm on unconstrained walking. The algorithm may also be adapted to a lighter protocol using only waist accelerometer signals and based on the same principle.

Another topic of interest is the choice of template to be used in the library. Several selection processes could be implemented in order to automatically adapt to any type of pathology and to optimize the performance of the algorithm.

## Figures and Tables

**Table 1:** Subjects’ characteristics. For the age, height and weight, the mean and the standard deviations are displayed.

Group	Number of Exercises	Number of Subjects	Sex (M/F)	Age (year)	Height (cm)	Weight (kg)
Healthy subjects	242	52	35/17	36.4 (20.6)	173.4 (10.8)	70.7 (12.2)
Orthopedic diseases	243	53	26/27	60.1 (19.3)	169.2 (10.2)	77.4 (16.8)
Neurological diseases	535	125	80/45	61.6 (13.2)	169.8 (8.7)	72.7 (15.5)
**Total**	1020	230	141/89	55.5 (19.6)	170.5 (9.7)	73.4 (15.3)

**Table 2:** Precision and recall scores for the template-based method and the Pan-Tompkins method. Means and standard deviations are displayed, along with *p*-values of the signed-rank Wilcoxon test.

	Template-Based Method		Pan-Tompkins		*p*-Value	
Group	Recall	Precision	Recall	Precision	Recall	Precision
Healthy subjects	99.31 (1.75)	99.13 (1.86)	99.14 (1.71)	97.09 (3.60)	0.286	1.57× 10^−19^
Orthopedic diseases	97.64 (2.73)	98.20 (3.93)	98.78 (2.09)	94.87 (5.09)	1.73 × 10^−8^	4.02 × 10^−23^
Neurological diseases	98.23 (3.42)	97.98 (3.33)	96.80 (3.52)	95.49 (4.55)	9.90 × 10^−24^	6.95 × 10^−42^
**Total**	98.34 (3.00)	98.30 (3.25)	97.82 (3.07)	95.72 (4.56)	7.49× 10^−7^	6.95× 10^−80^

**Table 3:** Precision and recall scores for the template-based method and the Pan-Tompkins method for different types of steps. Means and standard deviations are displayed.

	Template-Based Method		Pan-Tompkins	
Type of steps	Recall	Precision	Recall	Precision
Normal (33,764 steps)	99.58 (1.51)	99.04 (3.39)	95.59 (5.20)	98.86 (2.54)
Initiation (2040 steps)	96.37 (13.88)	97.75 (11.50)	95.59 (15.02)	95.93 (15.02)
Termination (2040 steps)	94.17 (17.37)	95.10 (15.83)	93.77 (16.95)	93.77 (16.95)
U-turn (2621 steps)	83.87 (27.88)	90.76 (23.96)	88.12 (23.45)	50.51 (30.23)

**Table 4:** Median absolute errors of ΔStart, ΔEnd, ΔDuration (in samples) for different types of subjects and steps. Results are displayed as ΔStart/ΔEnd/ΔDuration.

Type of Steps	Healthy Subjects	Orthopedic Diseases	Neurological Diseases
Normal	4/4/7	5/5/7	5/6/8
Initiation	5/6/6	6/5/7	6/7/11
Termination	5/6/10	6/7/9	6/8/10
U-turn	8/8/12	9/15/13	7/8/10
